# Endodontic Surgery of the Palatal Root of a Maxillary Molar Associated with Simultaneous Management of a Maxillary Sinus Lesion

**DOI:** 10.1155/2023/9180800

**Published:** 2023-07-12

**Authors:** Silvio Taschieri, Stefano Corbella, Luca Francetti, Alice Alberti, Benedetta Morandi

**Affiliations:** ^1^Department of Biomedical, Surgical and Dental Sciences, Università degli Studi di Milano, Milan 20123, Italy; ^2^IRCCS Istituto Ortopedico Galeazzi, Milan 20161, Italy; ^3^Institute of Dentistry, I. M. Sechenov First Moscow State Medical University, Moscow, Russia

## Abstract

This case report describes a particular application of endodontic microsurgery with a palatal approach in the presence of a radiopaque lesion inside the maxillary sinus. The patient presented complaining of pain related to the first maxillary molar and events of nasal obstruction and facial pain in the cheek and nasal area. The endodontic orthograde treatment and retreatment were done, respectively, 7 and 4 years earlier. The cone-beam computed tomography (CBCT) scan taken before the treatment showed two separate lesions: one associated with the palatine root of the molar and another one inside the maxillary sinus. The patient agreed to solve both problems in one surgical step: endodontic surgery of the palatine root with palatal access with the simultaneous asportation of a lesion from the maxillary sinus floor. Complete bone healing of the periapical area and the maxillary sinus was visualized on intra-oral radiographs, and CBCT was taken one year after the treatment. As far as the authors know, no one in literature has ever described this approach and solved in such a conservative way both the problems at the tooth and in the maxillary sinus.

## 1. Introduction

Over the years, the success rate of endodontic surgery has increased to 90% thanks to the improvement of surgical instruments, materials, and techniques [1, 2]. While traditional endodontic surgery performed with burs, amalgam root-end filling, and no or low magnification exceeded a success rate of 59%, this reached 90% with endodontic microsurgery (EMS), a modern technique that includes ultrasonic root-end preparation, IRM, Super-EBA or Mineral trioxide aggregate filling, and high magnification [1].

However, some conditions can complicate the surgical treatment, such as the presence of important anatomic structures and limited surgical access. For these reasons, the endodontic surgical management of maxillary molars can be considered one of the most challenging interventions [3]. In particular, the treatment of the palatine root due to the position of the greater palatine foramen, the proximity of the main vessels and nerves to the apexes, the depth of the palatal vault, the configuration of the zygomatic process of the maxilla, and the proximity to the maxillary sinus are some of the anatomical factors that must be considered before choosing the surgical procedure [4–6]. Moreover, according to Walton and Wallace, the maxillary sinus lies between the roots of the maxillary molars in 40% of cases [7], whereas for other authors the maxillary sinus floor can even be located between the roots in 94% of cases for the first molar and 81% of cases for the second molar [5]. In addition, several studies have shown that upper molars have the highest endodontic failure rate because an additional canal is often found in the mesiobuccal root and, less frequently, an extra C-shaped canal can be found in the palatine and distobuccal roots [8, 9].

Few studies have focused on the endodontic surgery of the upper molar teeth with a success rate that, depending on the type of surgery and the parameter used, such as a clinical parameter or two-dimensional (2D)/three-dimensional (3D) radiographic healing parameters, ranged between 44% and 88% [10–13]. However, none of them considered the teeth with a sub-analysis of the affected root.

In literature, two approaches have been described to reach and manage the palatal root depending on the presence of the maxillary sinus recess between the buccal and palatal roots and in consideration of the divergence between them: the transantral approach or the palatal approach [14–16].

Particularly, the transantral approach is indicated when the maxillary sinus floor has proximity with the palatal root apexes when its recess is located between the buccal and the palatal root or when the palatal root is inside it [14]. Whereas, the palatal approach is recommended when there is a risk of damage to the major palatine nerve and vessels. In both the transantral and the palatal approaches the perforation of the maxillary sinus and the iatrogenic sinusitis are complications that may occur and must be taken into consideration. Many authors, in different cadaveric and radiographic studies, have shown the proximity between the root apices of the maxillary posterior teeth and the sinus floor [17–19]. This can lead to accidental oroantral communication during various stages of the surgery [20]. Hauman et al. have shown that the invasion of a healthy maxillary sinus does not appear to cause a permanent alteration of its physiological function because the sinus mucosa regenerates five months after its surgical removal [21]. Another study has demonstrated that the perforation of the sinus membrane during a sinus lift procedure did not affect the result of bone grafting [22]. However, it is important to protect the sinus from the introduction of foreign bodies or bacteria present in the pathological dental elements that could cause acute or chronic sinusitis [21, 23].

Furthermore, some pathological conditions can affect the maxillary sinus, which can complicate the surgical approach of the posterior maxillary area, for example, the pseudocyst and mucous retention cyst. Both of them are self-limiting and benign lesions that originate inside the maxillary sinus due to the accumulation of liquids [24–26], and they are radiologically indistinguishable, as they both appear as a dome-shaped radiopaque lesion located at the floor of the maxillary sinus [24, 26]. The pseudo-cyst has no epithelial lining and is characterized by a thickening of the Schneidarian membrane due to the local retention of inflammatory exudation, whereas the mucous retention cyst is surrounded by a thin layer of epithelial cells and results from the obturation of a duct of the seromucous glands. Their finding is random and it usually occurs during routine radiographic assessment, since they are asymptomatic in the majority of patients [24, 28]. The treatment is not needed in the absence of clinical signs and symptoms, and in 30% of cases, they regress spontaneously. However, in 10% of cases, they can increase in volume and may lead to headache, periorbital or facial pain, nasal obstruction, and predispose to the development of recurrent rhinosinusitis. In such cases, surgical treatment is recommended [24, 27, 28].

This report described for the first time in literature a case where a palatal approach was used both for the management of a compromised palatal root of a maxillary first molar, and the asportation of a lesion from the maxillary sinus floor. In this way, two problems have been solved in one single and conservative surgical step.

## 2. Case Report

**Figure 1:**
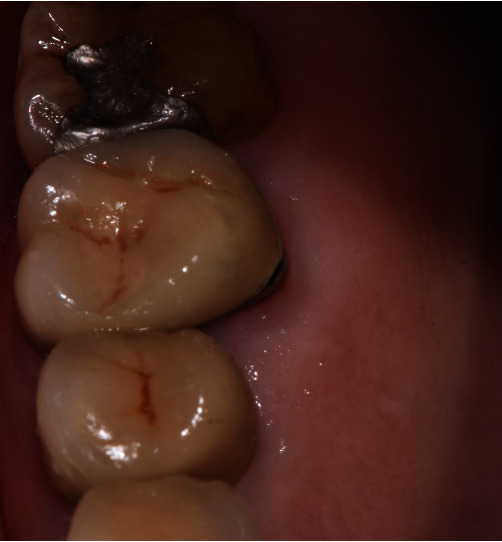
Palatal view of the right first upper molar.

**Figure 2:**
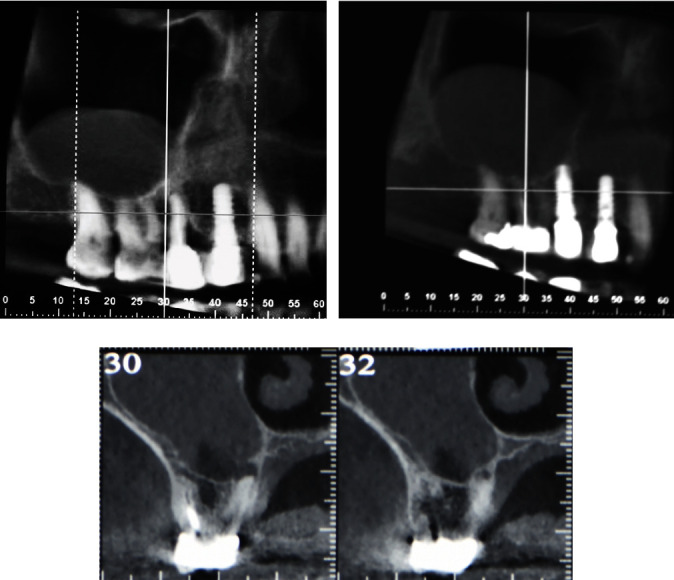
(a, b, and c) CBCT pre surgery showing the right first upper molar with a palatal radiopaque lesion associated with a cupoliform lesion inside the maxillary sinus.

A 75-year-old female patient, American Society of Anesthesiologists I, presented complaining of pain related to the right maxillary first molar and events of nasal obstruction, and facial pain in the cheek and nasal area. The patient had a history of non-surgical root canal treatment performed seven and four years earlier with an intact full-coverage crown. According to the patient, the tooth had been treated for deep caries that had caused necrosis of the tooth, and the cone-beam computed tomography (CBCT) requested from the first clinician had shown a cupuliform or “rising-sun” shape radiopaque lesion inside the maxillary sinus above the tooth. Clinical examination revealed no tooth mobility, no swelling, and no pain sensation to percussion and/or chewing (Figure 1). A periapical radiograph indicated an inadequate root canal filling and periapical radiolucency. The CBCT (3D Accuitomo 80, J. Morita, Kyoto, Japan) prescribed for planning the surgery showed a periapical low-intensity area associated with palatine root in contact with the maxillary sinus with the presence in the maxillary sinus of the cupuliform radiopaque lesion of increased size compared with the previous examination (Figures 2(a), 2(b), and 2(c)). Alternative treatments that included the extraction of the teeth or only the non-surgical endodontic re-treatment were discussed with the patient. The patient agreed to solve both the problem of the periradicular lesion with the EMS and the lesion inside the maxillary sinus with aspiration, and the patient signed an informed consent form.

### 2.1. Surgical Procedure

**Figure 3:**
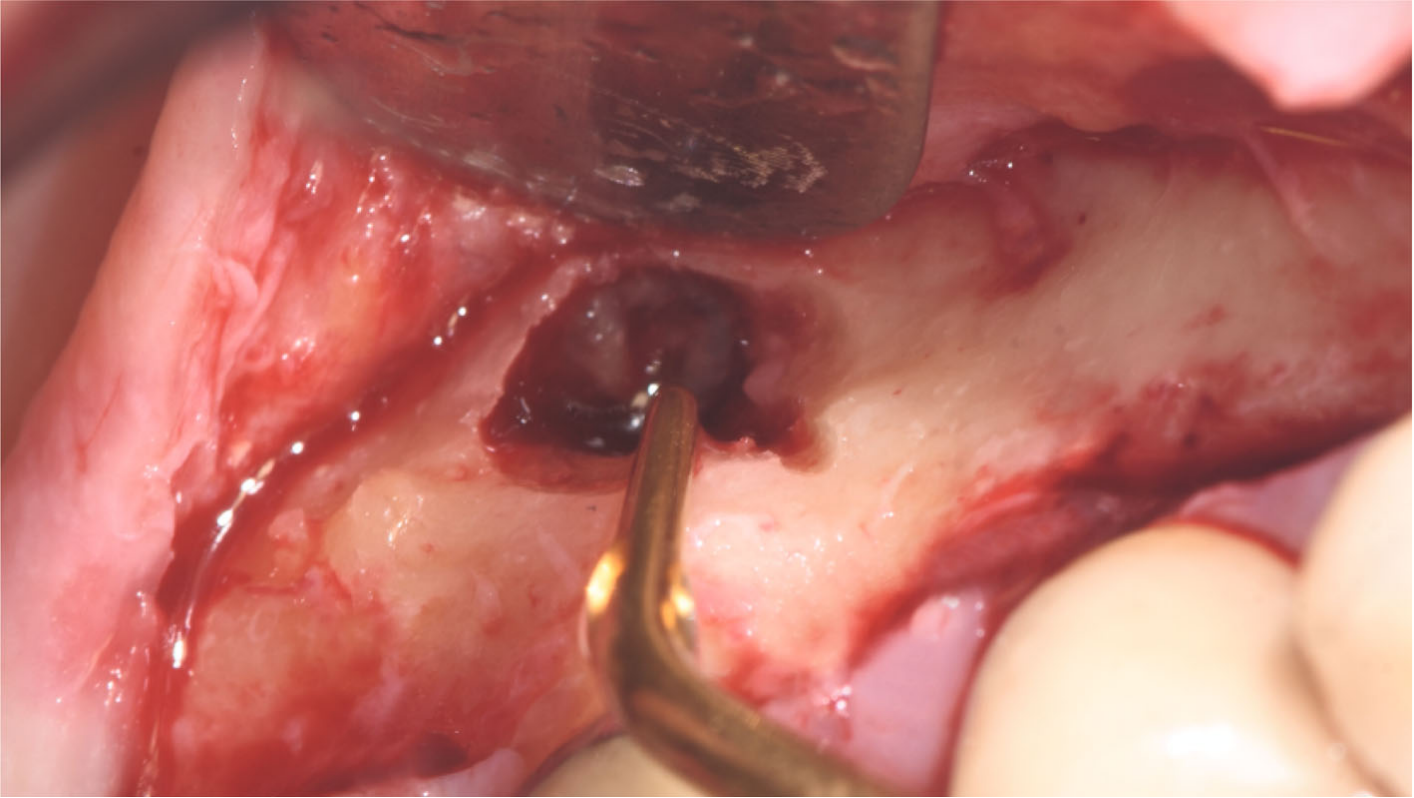
Retrograde preparation of the root performed with ultrasonic microtips.

**Figure 4:**
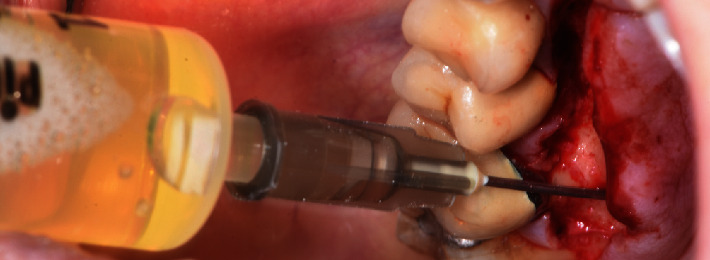
Aspiration of the liquid content inside the maxillary sinus with a syringe.

**Figure 5:**
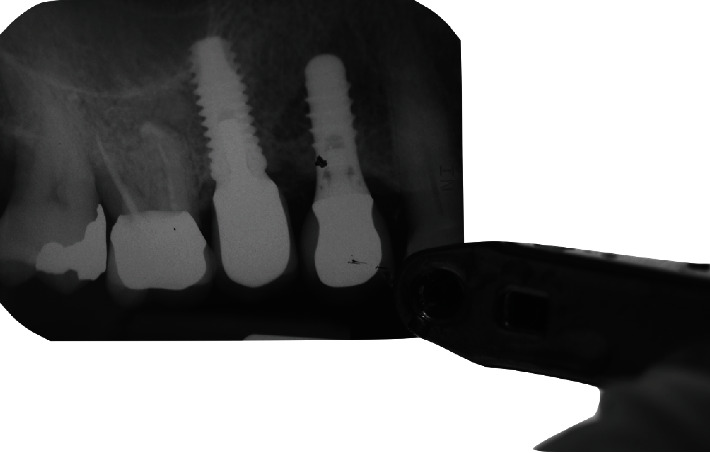
Post-operative periapical radiograph.

The modern microsurgical technique was performed using surgical loupes (5×). To reduce the number of bacteria in the surgical field, the patient was made to rinse with chlorhexidine digluconate (0.12%) before the surgical procedure. Local buccal and palatal anesthesia was gently administered in the maxillary right quadrant with articaine 4% and epinephrine 1 : 100,000. A full-thickness papilla preservation flap without vertical incision from the palatal right maxillary first premolar to the distal part of the second molar was elevated and retracted carefully during the surgical procedure and continuously irrigated with sterile saline solution. After flap elevation, bone was removed in the apical part of the palatine root using round burs under irrigation to expose the root apex. The exact point where to perform the osteotomy was calculated in the CBCT starting from the crown of the tooth and transferred in the mouth with a probe. After the complete removal of the lesion, the palatal root was resected 3 mm from the apex with a fluted fissure bur as perpendicular as possible (0°–15°) relative to the long axis of the root. The apex was extracted very delicately without damaging the maxillary sinus membrane to allow the management of the apical third in a safe situation, avoiding contamination of the maxillary sinus with apical residues or material for root canal obturation. The retrograde preparation of the root was performed using ultrasonic microtips (Endo Success Apical Surgery: AS3D, ACTEON Equipment—SATELEC, Merignac, France), to allow a parallel preparation (Figure 3). After adequate control of the hemostasis by local compression with sterile gauze impregnated with epinephrine, the prepared root-end was dried with paper points and sealed using Super EBA (SE; Harry J. Bosworth Co., Skokie, IL, USA). Afterward, the liquid content inside the lesion was sucked out completely with a syringe, emptying it (Figure 4). Finally, the flap was repositioned and closed using Ethilon 5-0 monofilament non-absorbable sutures (Monosof; Covidien LLC, Mansfield, MA, USA). A postoperative periapical radiograph was taken (Figure 5), and the patient was instructed to rinse twice daily with 15 mL chlorhexidine digluconate 0.2% up to 10 days after surgery, and to take amoxicillin and clavulanic acid 1 g, *N*-acetylcysteine aerosol up to 6 days after surgery, and anti-inflammatory nonsteroidal drugs (ibuprofen 400 mg) twice a day for two days after surgery for swelling and pain control. Sutures were removed 7 days after surgery.

### 2.2. Follow-Up

**Figure 6:**
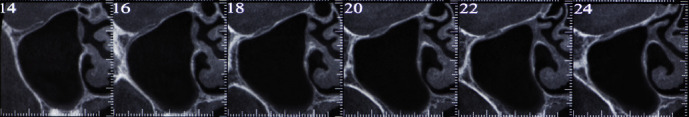
CBCT showing a complete remission of the sinus lesion.

At the 6-month follow-up, a clinical assessment was performed, and a periapical radiograph was taken, whereas, at the 12-month follow-up, both periapical radiograph and CBCT were analyzed. The patient came to a recall appointment at 6 and 12 months after surgery with no symptoms. Soft tissue examination showed no sign of a gingival recession and revealed healthy periodontal tissue with normal probing around the tooth. The periapical radiographs at 6 and 12 months revealed no more apical radiolucency. At the last appointment, the CBCT showed a complete remission of either the periapical lesion or the lesion inside the maxillary sinus (Figure 6). The palatal root was categorized as complete healing based on both 2D and 3D radiographic criteria for success [29, 30] as it has been detected the complete hard tissue fill of the former lesion and formation of an intact cortical plane.

## 3. Discussion

Our case report showed a particular application of EMS with a palatal approach in the presence of a radiopaque lesion in the maxillary sinus. Wallace proposed a trans-antral approach to reach the palatine root of the maxillary molars [14–16]; this technique has some main indications: when the apices of the palatal root are close to the maxillary sinus or it penetrates it, when the maxillary sinus recess is located between the buccal and palatal roots [14], and when all the roots are involved to avoid to raise two flaps and performing two osteotomies, one in the buccal and another one in the palatal aspect. However, this technique can be associated with multiple technical difficulties, such as excessive bleeding, poor visibility, and the complexity to do a sinus lift with the presence of the roots. This can lead to a large sinus perforation, which can sometimes turn into sinusitis [3, 31]. A different approach to reach the site is by raising a palatal flap and addressing the root through a palatal approach. This technique can either be associated with several potential complications, such as the difficulty in positioning and the lack of direct visualization, or the need to raise two flaps if the buccal roots also require intervention and the risk of injury to the greater palatine nerve and vessels during flap reflection, retraction, or osteotomy [4]. In the clinical case presented a palatal approach was performed to be as conservative as possible: the only root with an apical lesion was the palatal, the palatine nerve and vessel were not involved in the flap, and the palatal cortical plate was perforated. It has also been shown by Lee et al. that the palatal approach to reach the palatine root for apical surgery has a high success rate with low complications [32]. The tomographic evaluation was fundamental in determining, which surgical approach to perform. Several studies have shown that the CBCT should be considered the imaging modality of choice for presurgical treatment planning of endodontic surgery, especially when vital anatomic structures are involved in the procedure [33–35].

After performing the EMS and cleaning the cavity, the liquid content in the maxillary sinus lesion was aspirated with a syringe.

It had been described in the literature that the mucous retention cyst or the pseudocyst inside the maxillary sinus are common lesions that in most cases remain in the sinus without any symptoms and for that reason, surgical intervention is often not necessary [26, 36]. However, in 10% of the cases, these lesions tend to increase in volume over time and can become dangerous and increasing the episode of sinusitis and headache [24].

In this case, the previous CBCT done years earlier was compared with the one required for surgery. It was clear that the lesion had increased in volume. This dimensional change, in addition to the symptoms described by the patient attributable to sinusitis, made us decide to intervene surgically taking advantage of the surgical access made for the endodontic surgery. In this way, we could solve both the apical and sinus pathology in a single surgical session. To manage the lesion inside the maxillary sinus, the procedure first described by Maiorana et al. [37] and Torretta et al. [38] in patients with maxillary cysts who had to undergo to sinus lift procedure was considered. They had reduced the volume of the cyst and therefore, the tension of the membrane by suctioning the liquid of the lesion with a fine needle inserted through the sinus membrane. Following this procedure, after completing the endodontic surgery procedure and having gently cleaned and delicately removed all the granulation tissue present in the cavity, we sucked the liquid, completely emptying the sinus and returning it to a normal physiological state. An alternative approach could be the one described by Felisati et al. [39] who have proposed a single surgical session combining an intraoral approach with transnasal functional endoscopic sinus surgery. Even if this can be considered an ideal solution because it can correct some anatomical problems, such as septa deviations, concha bullosa, or a non-patent or partially patent ostium, and can remove all the sinus cyst, it presents some limitations, such as it requires the combination of ENT surgeons and oral maxilla-facial surgeons together, the patient needs a hospitalization because the treatment has to be performed under general anesthesia resulting in a significant increase in cost, operative time, and postoperative morbidity. In the literature, it is still controversial whether the lesions should be aspirated or removed [40].

## 4. Conclusions

Data are lacking about if aspiration without enucleation may expose the patient to an increased risk of relapse. As far as we know, no one has ever described the EMS of the palatal root of a maxillary molar through a palatal approach associated with the suction of a lesion inside the maxillary sinus. With this conservative approach, we have successfully solved both problems. Furthermore, studies are needed to evaluate the effectiveness of this surgical technique.

## Data Availability

No data were available.
